# Unilateral Variation of the Left Ascending Pharyngeal Artery: A Case Report

**DOI:** 10.7759/cureus.69740

**Published:** 2024-09-19

**Authors:** Maurice N Maglasang, Lokesh A Coomar, Meadow Campbell

**Affiliations:** 1 Anatomy, New York Institute of Technology College of Osteopathic Medicine at Arkansas State University, Jonesboro, USA; 2 Department of Surgery, Center for Anatomical Science and Education, Saint Louis University School of Medicine, St. Louis, USA

**Keywords:** anatomical variation, ascending pharyngeal artery, common carotid artery, external carotid artery, head and neck vessels, internal carotid artery, vasculature

## Abstract

During routine cadaveric dissection of a 77-year-old male, a series of unique arterial variations were observed within the left anterior cervical region. Firstly, the ascending pharyngeal artery (APA) was observed branching from the common carotid artery (CCA), between the internal (ICA) and external carotid (ECA) arteries. The APA continued superiorly without branching, as is typical for APA. Secondly, a common arterial trunk was observed emerging from the posterior cervical segment of the left ICA. As the common trunk continued superiorly, it bifurcated into two distinct branches. The first branch terminated as several muscular branches (MB) feeding the salpingopharyngeus and superior constrictor muscles. The second branch, a suspected neuromeningeal trunk (NMT), continued superiorly and terminated within the jugular foramen. We believe this individual presented with atypical branching of the APA system. The course of both the muscular branch and the neuromeningeal trunk were novel in that they both originated from a common trunk off the ICA. Both variations could have pathologic and clinical implications.

## Introduction

The ascending pharyngeal artery (APA) has been described as the smallest branch of the external carotid artery (ECA) [1]. Classically, it is the second most inferior branch to originate from the ECA, arising on its posteromedial aspect, distal to the ECA’s origin from the common carotid artery (CCA) [2,3]. The APA ascends anterior to the longus capitis muscle and medial to the styloglossus and stylopharyngeus muscles. The musculospinal artery is the most proximal branch to originate from the APA, prior to the APA bifurcating into the pharyngeal and neuromeningeal arterial trunks [2,4,5]. The anteriorly placed pharyngeal trunk continues extracranially. The neuromeningeal trunk continues posteriorly, entering the cranial vault via the foramen magnum [2]. The musculospinal artery anastomoses with the vertebral artery at the level between the second and third cervical vertebrae. It can also anastomose with the ascending cervical artery at the level of the third cervical vertebrae [2]. This artery supplies the spinal accessory nerve as well as the superior cervical ganglion [2]. The pharyngeal trunk of the APA gives off the superior, middle, and inferior pharyngeal arteries. These three branches supply the pharyngeal submucosal spaces [2]. Additionally, a branch arising from either the superior or middle pharyngeal artery perfuses the eustachian tube [2]. The superior pharyngeal artery gives off a small branch that runs with the ICA in the foramen lacerum. This branch may anastomose with the ICA via the inferolateral trunk and/or the recurrent artery of the foramen lacerum [2]. The middle pharyngeal artery can also anastomose with the descending palatine branch of the maxillary artery [2]. The neuromeningeal trunk of the APA further divides into the hypoglossal branch, the jugular branch, and the clival branch. The hypoglossal branch passes through the hypoglossal canal, accompanied by the hypoglossal nerve. This branch supplies the hypoglossal nerve as well as the meninges of the posterior cranial fossa, specifically the dura surrounding the foramen magnum and clivus [2,4]. The hypoglossal branch also supplies the first three cervical nerve roots through a series of smaller branches [4]. This artery may anastomose with the internal carotid artery (ICA) or the vertebral artery [2,4]. Occasionally, the hypoglossal branch can arise from the vertebral artery rather than the neuromeningeal trunk of the APA [4]. The jugular branch passes through the posterior cranial fossa and enters the jugular foramen, accompanied by the glossopharyngeal, vagus, and accessory nerves, all of which are supplied by it [2,6]. Small branches of the jugular branch supply the meninges of the internal auditory canal, the dura associated with the inferior petrosal and sigmoid sinuses, and the abducens nerve, specifically before it enters Dorello’s canal [2-4]. These small branches allow the jugular branch to anastomose with the mastoid branch of the occipital artery, as well as the posterior meningeal branch of the vertebral artery [4,6,7]. Additionally, branches of the jugular branch anastomose with the petrosquamosal branch of the middle meningeal artery [1]. The clival branch of the neuromeningeal trunk supplies the clivus and associated region [2]. It may anastomose with the medial clival branch of the inferior hypophyseal artery as well as the anterior meningeal artery, which arises from the vertebral artery [4]. The inferior tympanic artery commonly arises as a branch of the proximal neuromeningeal trunk. However, it can originate as a separate branch of the APA between the pharyngeal and neuromeningeal trunks [2]. It travels with the glossopharyngeal nerve and anastomoses with the caroticotympanic branch of the ICA. This anastomosis can perfuse the glossopharyngeal nerve [2]. Like the branches of the jugular branch of the neuromeningeal trunk, the inferior tympanic artery can also anastomose with the petrosquamosal branch of the middle meningeal artery. The inferior tympanic artery anastomoses with the stylomastoid artery, a branch of the posterior auricular artery, supplying the facial nerve in the process [2,8]. Finally, the neuromeningeal trunk may give off a posterior descending branch proximal to the inferior tympanic artery, which anastomoses with the vertebral artery through the odontoid arch system. This arterial system surrounds the dens of the second cervical vertebra and is more concentrated around the neck of the dens [2,9]. Unlike the classical presentation described above, this report describes a novel series of ICA and APA arterial variations not observed previously. This unique set of variations presents an opportunity to investigate the arterial supply of both the pharynx and posterior cranial fossa.

## Case presentation

**Figure 1:**
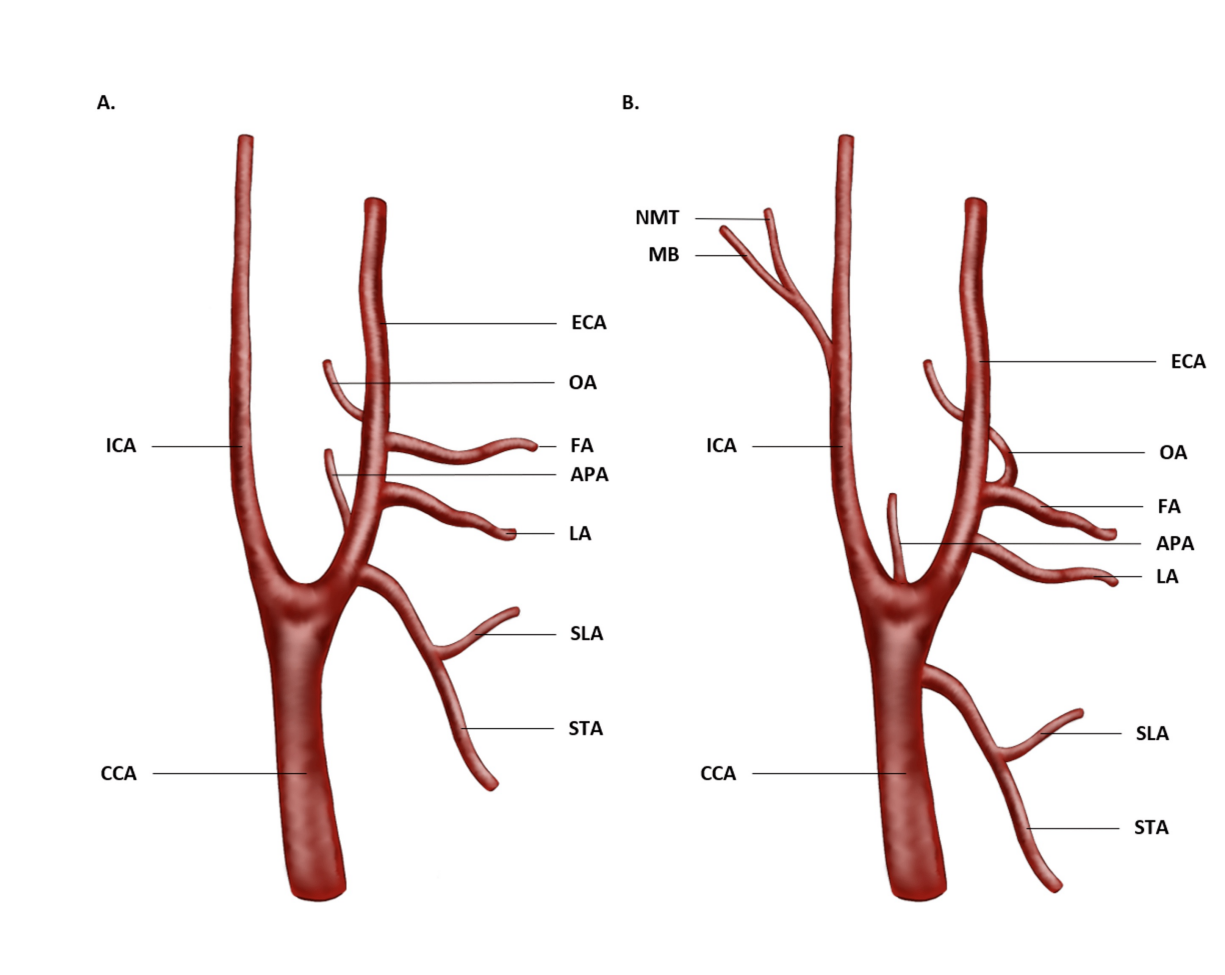
A comparison of the normal branching pattern in the left common carotid arterial system (A) to the variations observed (B). Normal branching of the ECA (A) is depicted, including the STA, APA, LA, FA, and the OA, as well as the SLA. The described variations in this case (B) include the STA originating from CCA, APA from the CCA, OA from FA, and the muscular pharyngeal trunk (MB) and neuromeningeal trunk (NMT) arising from a common APA trunk from the ICA. (CCA: Common carotid artery, ECA: External carotid artery, ICA: Internal carotid artery, STA: Superior thyroid artery, SLA: Superior laryngeal artery, APA: Ascending pharyngeal artery, LA: Lingual artery, FA: Facial artery, OA: Occipital artery, MB: pharyngeal muscular trunk, NMT: neuromeningeal trunk).

**Figure 2:**
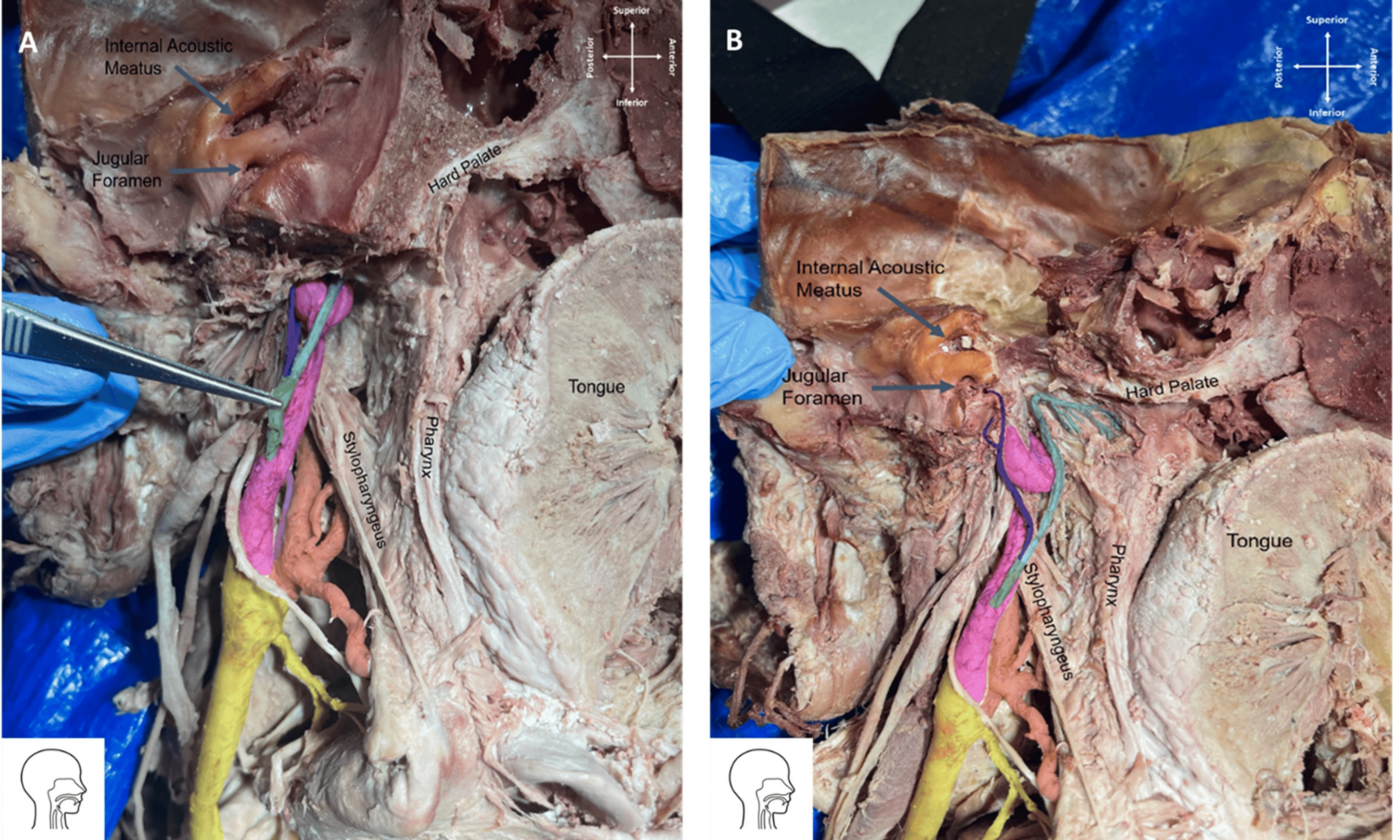
(A) Branching pattern of the arteries stemming from the left carotid vessels within the cervical region (B) and more superiorly towards the posterior cranial fossa. There are three branches arising from the left CCA (yellow): the left ECA (orange), the left ICA (pink), and the 1st APA (light violet). The 2nd APA (green) stems from the left ICA and gives off an AA (cyan) and AB (dark violet). (ECA: External carotid artery, ICA: Internal carotid artery, APA: ascending pharyngeal artery).

A 77-year-old male donor was received through the Saint Louis University Gift of Body Program of the Center for Anatomical Science and Education (CASE) with signed informed consent from the donor. The CASE gift body program abides by all the rules set forth by the Uniform Anatomical Gift Act. Additionally, in accordance with the Declaration of Helsinki (WMA, 2013), informed consent was obtained from all participants, ensuring they were fully aware of the study's purpose, procedures, risks, and benefits. During routine dissection, the bisected head of the donor was disarticulated from the first cervical vertebrae and reflected anteriorly to separate the neck viscera from the prevertebral region of the neck. As the right carotid sheath was cleared to examine its contents, the anticipated carotid branching pattern was observed. The right CCA bifurcated into an ECA and ICA at the level between the third and fourth cervical vertebrae. The right ICA had no visible branches through its course towards the carotid canal. The right ECA artery demonstrated a typical branching pattern as well. The contents of the left carotid sheath were also examined. The left CCA bifurcated into the ECA and ICA at roughly the same level as its right counterpart, between the third and fourth cervical vertebrae. In addition, another artery was observed arising from the CCA directly in between the ECA and ICA. No branching was observed on this vessel. We concluded that it was most likely the left APA due to its anterosuperior course towards the sphenopalatine region and the fact that the left APA was absent from the left ECA. Apart from the aforementioned APA branching, additional branching was observed emerging from the left ICA. A common trunk emerged from the posterior surface of the left ICA, approximately 27 mm above the left CCA bifurcation within the ICA’s cervical segment. This common trunk then bifurcated into two distinct branches (Figures 1, 2).

Initially, we believed that both anomalous branches accompanied the left ICA into the cranial vault through the carotid canal opening. However, upon removing a portion of the petrous temporal bone containing the carotid canal, it was revealed that neither branch passed through the canal. Instead, the first branch, identified as a direct branch originating off the pharyngeal trunk of the left APA, was observed traveling towards the posterior aspect of the torus tubaris. The nasopharyngeal mucosa was reflected to expose the full extent of the artery. The artery was observed terminating as multiple muscular branches for the salpingopharyngeus muscle. The second branch, identified as the neuromeningeal trunk, terminated within the jugular foramen. It was concluded that the common trunk emerging from the left ICA was an additional left APA. It is worth noting that the neuromeningeal trunk may have had branches continuing superiorly past the foramen magnum. However, since the brain was extracted prior to this dissection, these branches were unintentionally excised.

## Discussion

While variations within the carotid arterial system are not uncommon, the series of variations observed in this case has not been reported in the literature. However, such variations within the anterior cervical region occur during embryological development. In the third week of gestation, the aorta begins to develop [10]. As development progresses, six pairs of aortic arches are formed between the dorsal and ventral aortae [10]. The ECA develops from the first pair of arches, while the ICA and CCA develop from the third pair of arches [11]. When remnants of the first pair are connected directly to the ICA or CCA instead of the ECA, variations are likely to form [11]. The most observed APA variations are when they arise from either the extracranial portion of the ICA or the occipital artery [12]. Additionally, evidence suggests that when present, the meningeal branch of the APA occurs more frequently on the right side of the body [13]. In this case report, the variation was observed on the left side.

The unilateral presence of two APAs could have a multitude of clinical and pathological implications. A patient with a complex anatomical variant of the APA such as this may require a more invasive treatment option such as open surgical resection of an aneurysm instead of less invasive procedures such as endovascular management with coil embolization [14]. More invasive surgical procedures and CT angiography are needed to treat aneurysms arising from complex anatomical variants. Additionally, to treat certain head and neck cancers, intraarterial embolization is often utilized as an effective treatment option [15]. Superselective catheterization of the APA is needed to target cancers of the palate [16,17]. Having two APAs may require angiographic intervention in order to place the catheter in the appropriate vessel. The APA has also been cited as providing blood supply to several oncological structures around the jugular foramen, such as meningiomas, neuroendocrine paragangliomas, and giant cell tumors [2]. Having two ascending pharyngeal arteries on one side may result in an increased blood supply to these structures, which could potentially have implications for their rate of growth and metastasis [18,19].

## Conclusions

The current case presents a unique set of arterial variations within the anterior cervical region. The case includes a unilateral anomalous arterial branching pattern contributing to the left APA system. These variations include the APA branching from the common carotid artery, between the ICA and ECA, as well as two accessory arteries originating from the ICA. The traveling pattern of the first branch was consistent with the muscular pharyngeal trunk of the APA, whereas that of the second branch was consistent with the neuromeningeal trunk of the APA. This series of variations within the APA system has yet to be described in the literature. Furthermore, documentation of unique APA variations is critical due to APA’s extensive intracranial and extracranial anastomoses, which can have implications in oncology and surgical interventions.
